# Executive function and processing speed in Brazilian HIV-infected children and adolescents

**DOI:** 10.1590/S1980-57642014DN81000006

**Published:** 2014

**Authors:** Vitor Geraldi Haase, Nelsa Carol Nicolau, Virgínia Nunes Viana, Gustavo de Val Barreto, Jorge Andrade Pinto

**Affiliations:** 1 Faculdade de Filosofia e Ciências Humanas - Universidade Federal de Minas Gerais, Minas Gerais, Brasil.; 2 Faculdade de Medicina, Programa de Pós-Graduação em Saúde da Criança e do Adolescente, Universidade Federal de Minas Gerais, Brasil.; 3 Instituto de Ciências Biológicas - Pós-Graduação em Neurociências, Universidade Federal de Minas Gerais, Brasil.; 4 FEAD - Minas; 5 Centro Universitário UNA.

**Keywords:** HIV, AIDS, neuropsychology, executive function, processing speed

## Abstract

**Background:**

Cognitive disorders in infants and children who are vertically infected with human immunodeficiency virus (HIV) have been recognized since the inception of the epidemic.

**Objective:**

The present study investigated neuropsychological performance in a cohort of vertically infected Brazilian children and adolescents who underwent antiretroviral therapy. The neuropsychological tasks were designed to evaluate executive function and processing speed.

**Methods:**

Children and adolescents were recruited at a major research and treatment reference center for human immunodeficiency virus/acquired immunodeficiency syndrome (HIV) in Minas Gerais, Brazil. Forty-one individuals aged 5 to 17 years were enrolled. Twelve were mildly symptomatic (HIV-infected group, Centers for Disease Control and Prevention [CDC] class A or B), and 29 had advanced clinical disease (AIDS group, CDC class C).

**Results:**

The results showed that HIV-infected children and adolescents exhibited lower performance on neuropsychological tasks than sociodemographically comparable, typically developing controls. Motor and cognitive processing speed and executive function appeared to be the most discriminative domains.

**Conclusion:**

HIV-infected individuals with more-advanced disease stages exhibited lower performance levels and had greater performance heterogeneity on neuropsychological tasks. Thus, the observed neuropsychological impairments, although more pronounced in participants with more advanced stages of the disease, did not correlate with the variable used (CDC stage).

## Abstract (pt)

**Introdução:**

Comprometimentos cognitivos em crianças e adolescentes verticalmente afetadas por HIV são reconhecidos desde do início da epidemia.

**Objetivos:**

O presente estudo investigou o desempenho neuropsicológico em uma coorte de crianças e adolescentes verticalmente infectadas por HIV e que fazem uso da terapia antirretroviral no Brasil. As tarefas neuropsicológicas foram selecionadas para avaliar, principalmente, funções executivas e velocidade de processamento.

**Métodos:**

As crianças e adolescentes foram recrutados em um grande centro de referência de pesquisa e tratamento para o vírus da imunodeficiência humana / síndrome da imunodeficiência adquirida (HIV / AIDS), em Minas Gerais, Brasil. Quarenta e um indivíduos com idade entre 5 e 17 anos foram selecionados. Doze estavam com sintomas leves (grupo infectado pelo HIV, Centros para Controle e Prevenção de Doenças [CDC] Classe A ou B), e 29 tiveram a doença clínica avançada (grupo AIDS, CDC classe C).

**Resultados:**

Os resultados mostraram que as crianças e adolescentes infectados pelo HIV apresentaram menor desempenho em tarefas neuropsicológicas quando comparadas com o grupo controle pareado socio-demograficamente. Habilidades motoras, velocidade de processamento e função executiva são os domínios mais discriminativos.

**Conclusão:**

Indivíduos infectados pelo HIV com estágios da doença mais avançados apresentaram níveis mais baixos de desempenho e tiveram maior heterogeneidade no desempenho em tarefas neuropsicológicas. Entretanto, os prejuízos neuropsicológicos observados, apesar de se mostrarem mais pronunciados com estágios mais avançados da doença, não se correlacionaram com a variável utilizada (estágio do CDC).

## INTRODUCTION

Cognitive disorders in infants and children who are vertically infected with human immunodeficiency virus (HIV) have been recognized since the inception of the epidemic. Most notable have been the early manifestation of compromised central nervous system function, a high incidence of severely impairing encephalopathy characterized by brain atrophy, calcifications, spasticity, and mental retardation, and the high mortality associated with the disease.^1^ The adoption of mother-to-infant transmission prevention measures^2^ and introduction and widespread access to antiretroviral therapy^3^ in the mid-1990s in developed countries and some developing countries, such as Brazil,^4,5^ represent a radical epidemiological transition. Antiretroviral therapy consists of the use of a set of drugs to treat retrovirus infections, especially HIV. Highly active antiretroviral therapy is known as HAART. As the number of new vertically transmitted cases decreases and children and adolescents survive longer and approach adulthood, the focus of care turns to treatment adherence, drug resistance, quality of life, and disease-associated morbidities, such as cognitive and behavioral disorders.^6^ Neuropsychologically, the most relevant transition is related to the decreasing incidence of HIV-associated encephalopathy and increasing incidence of minor cognitive and behavioral disorders frequently observed in adults.^7^

There is a lack of information about more subtle forms of neuropsychological impairment in older youths with HIV/acquired immunodeficiency syndrome (AIDS) during adolescence. A systematic review by^8^ selected 54 empirical studies that investigated neurodevelopment in the pediatric population. Thirty-one studies focused on infancy and preschool age. Thirty-four studies were restricted to psychopathological manifestations or lacked a neuropsychological clinical-anatomical assessment framework and used only global measures of development, intelligence, and school achievement. Five studies investigated the cognitive and behavioral aspects of HIV/AIDS associated with blood product transfusion in children. Finally, eight studies employed a neuropsychological structural-functional assessment framework, four of which were published before the effective treatment era.^9-12^ In addition to the four neuropsychological studies reported by,^8^ we were able to identify a fifth study that was published afterward.^13^

All five post-effective treatment era antiretroviral therapy neuropsychological studies used cross-sectional designs and several cognitive measures that covered different performance domains^13-15^ or analyzed intelligence test data in a manner that makes structural-functional interpretations viable.^16,17^ With the exception of participants in the study^16^ who showed no neuropsychological impairment, the results of the other studies converged, showing that psychomotor and cognitive processing speed, visuospatial abilities, and executive function were the cognitive domains most frequently impaired in individuals who presented with AIDS.

Visuospatial and visuomotor disorders in the pediatric population are frequently observed in conditions such as shunted congenital hydrocephalus, in which damage occurs to subcortical white matter tracts.^18^ Deficits in psychomotor/cognitive speed and executive function are ascribed to fronto-striatal connection damage, constituting the so-called subcortical white-matter pattern of impairment.^19^

Some anatomical evidence indicates that fronto-striatal white matter may be a privileged locus of impairment in HIV-infected individuals, both in adults^20^ and children.^21^ A sophisticated investigation of 300 infected adults that utilized factor- and cluster-analytic methods confirmed the existence of isolated executive function and processing speed patterns of impairment.^22^ The overall picture, however, is more complex. Two subcortical patterns of impairment coexist with other forms of neuropsychological compromise, proportionally mixing deficits in verbal episodic and visuospatial memories with deficits in executive function, working memory, and processing speed.

The present study investigated neuropsychological performance in a cohort of vertically infected Brazilian children and adolescents who underwent HAART regimens. Specifically, we investigated the hypotheses that^1^ HIV-infected children and adolescents exhibit lower performance on neuropsychological tasks than sociodemographically comparable, typically developing controls,^2^ that motor and cognitive processing speed and executive function are the most discriminative domains, and^3^ that HIV-infected individuals in more-advanced CDC disease stages exhibit lower performance levels or have greater performance heterogeneity on neuropsychological tasks.

## METHODS

Children and adolescents with vertically acquired HIV/AIDS infection were recruited at a major research and treatment reference center for HIV/AIDS in Minas Gerais, Brazil, operating at the University Hospital, Federal University of Minas Gerais in Belo Horizonte. Forty-one individuals aged between 5 and 17 years were enrolled. Twelve were mildly symptomatic (HIV-infected group, CDC class A or B), and 29 had advanced clinical disease (AIDS group, CDC class C). The mean ages of the HIV-infected group and AIDS group were 11.17 years (SD=2.98 years) and 10.86 years (SD=2.97 years), respectively. The descriptive data sample is shown in Table 1. No indication of peripheral neuropathy that could potentially influence performance was found in the clinical group. The exclusion criteria adopted was delay in development, mental illness, mental retardation, learning disabilities, physical or sensory impairments, chronic disease or motor disabilities.

**Table 1:** Descriptive data sample by group.

		Control	HIV	Aids
School	Public	72	12	28
	Private	10	0	1
Sex	Male	38	6	16
	Female	44	6	13
Age (m ; sd)		10.95 ; 2.92	11.17 ; 2.98	10.86 ; 2.97
Age range		5-17	8-17	5-17

Subjects for the comparison HIV-uninfected group were recruited in Belo Horizonte, the state capital with 2.4 million inhabitants. The control group was composed of 82 typical controls, pseudo-matched to the HIV/AIDS patients with regard to age, gender, school type and z-scores on the intelligence test. They were recruited and assessed randomly in public and private schools in Belo Horizonte. The proportion used for the pairing was 2:1. The results of the intelligence test were converted to z-scores because the Brazilian versions of the Raven test are not the same for the entire age group (5 to 17 years).

General fluid intelligence was assessed with age-appropriate, Brazilian-validated versions of Raven's matrices.^23^ Visuoconstructional abilities were assessed using a modified Bender Gestalt Test,^24^ in which the child has to copy nine geometric figures of increasing complexity.

**Executive function.** The test selection for the neuropsychological assessment of executive function was inspired by the trifactorial model of executive function.^25,26^ Latent variable analyses showed that executive function may be described by an overarching general factor and three hierarchically subordinated and partially dissociable components.^27^ The chosen tasks may not be considered pure measures of each component, but performance on the backward Digit-Span task is taken as an index of working memory, and the Stroop paradigm assesses inhibition. Test selection was biased toward easier tasks that could assess individuals within broader age and performance level ranges. The task battery was previously validated in children of preschool age.^28^ Also, no adjustments in the tests used in older subjects were performed. The main objective was to describe which tasks have the greatest potential to discriminate the performance of the participants of the clinical group according to the parameters of the disease. The following executive function tasks were used:

*Digit-Span task* - The direct and reverse Digit-Span tasks were used to assess working memory according to procedures of the Brazilian Wechsler Intelligence Scale for Children, 3rd edition.^29^ Total scores were used as dependent measures.

*Day-night Stroop test* - The Day-night Stroop test^30^ was used to assess the monitoring/error detection component of executive function.

*Semantic word fluency task* - The semantic fluency task was adapted from.^31^

The children were asked to name as many exemplars from a given category as fast as possible in 60 s, taking care not to repeat any names. The categories included animals, body parts, and food.

*Visual Search task* - Two visual search tasks were used to tap selective attention. The object search task was adapted from,^31^ and the square search task was adapted from.^32^ In both tasks, the stimuli consisted of line drawings and were presented on A4 sheets of paper.

*Tower of Hanoi task* - The Tower of Hanoi task was used as a measure of more complex executive function, tapping planning, strategy use, and set-shifting abilities. The procedures were adapted from.^33^

*List Discrimination Test* - The List Discrimination Test was adapted by^34^ from procedures described by.^35^ The test assesses both recognition memory and a form of recency or temporal order memory related to the dor-solateral prefrontal region.^36^

**Processing speed.** Both adult^37^ and pediatric^13,17^ studies suggested that processing speed is an important component of neuropsychological impairment in HIV/AIDS. A series of simpler processing speed measures was considered necessary to determine whether deficits originate from processing speed *per se* or from more complex functions and abilities. Measures in the manual and articulatory response modes were also considered necessary.

*Nine-hole Peg Test (9-HPT)* - The 9-HPT assesses the time required to take nine pegs, one by one, from a concavity, place them in respective holes, remove them, and return them to the original holder. Two trials are conducted for each hand, beginning with the dominant hand. The 9-HPT has become standard in the context of multiple sclerosis,^38^ but standardization studies have been conducted with children and adolescents.^39,40^

*Simple articulatory speed* - This was assessed by determining the mean time to complete three tasks. In the first task, the color naming phase of the Victoria Stroop test was used according to.^41^ Articulatory speed was also assessed by measuring the time required to count from 1 to 6 in forward and reverse orders. Finally, the children were asked to name as fast as possible a series of line drawings displayed on an A4 sheet of paper (moon, cup, ribbon, and candy), pseudorandomly repeated in a 5 column × 3 row matrix.

The study procedures were approved by the UFMG Institutional Review Board. Participation required an informed consent form signed by the children's parents or legal guardians. Participants in the clinical samples (HIV and AIDS) were assessed at their regular clinic visits. The testing of the clinical participants was conducted by a trained psychologist. The testing of typical participants was conducted by a psychologist and group of five undergraduate psychology students who were trained in the study procedures. Testing procedures occurred in the participating schools.

**Statistical methods.** Where allowed by the test design, a series of Cronbach's alpha coefficients were estimated to separately assess the scores' internal consistency in the clinical group and control pool. All alpha values were situated above 0.7.

In addition to descriptive statistics, ANOVA was used to compare the performance of the three groups (controls, HIV and AIDS). Receiver Operating Characteristic (ROC) curve analysis was employed to investigate the discriminative power of neuropsychological tests used as reference based on only two groups, control and clinical (HIV and AIDS).

## RESULTS

**Group comparisons.** Central tendency and dispersion estimates for the neuropsychological test scores in each group are shown in Table 2. Global intergroup comparisons using analysis of variance (ANOVA) revealed significant differences in major neuropsychological parameters. A Bonferroni correction for three multiple comparisons was used. Most of the *post hoc* comparisons were significant, with the exception of the Tower of Hanoi - total number of moves and number of rule violations, respectively (F=2.93, p=0.061; F=1.32, p=0.43), List Discrimination Test- recognition (F=2.705, p=0.284), abstract Stroop (F=2.206, p=0.124),and semantic word fluency-perseverative errors (F=0.459, p=1.00; Table 2).

**Table 2:** Neuropsychological test score distribution in control, HIV and AIDS groups. ANOVA in the neuropsychological tests, F values, p level of significance and post hoc tests.

Neuropsychological tests	Controls				HIV				AIDS			F	p	*Post hoc*
	n	Mean	SD		n	Mean	SD		n	Mean	SD			
Raven z score	82	-0.786	1.76		12	-0.80	1.58		29	-1.71	1.90	2.982	0.053	-
9-HPT - Total time for dominant hands	82	19295.98	3417.40		12	21932.92	4868.38		29	22983.57	6356.39	8,159	0.001	b
9-HPT - Total time for nondominant hands*	82	21030.12	4588.71		12	22133.33	4344.95		29	26072.32	9866.51	6.957	0.001	b
9-HPT - Mean total time for both hands*	82	20163.05	3501.41		12	22033.13	4335.13		29	24527.95	7795.62	8.460	0.001	b
Color naming - Total time*	82	77353.79	22946.58		12	103113.85	44046.15		29	116231.17	48011.53	16.92	0.001	b
Picture naming - Total time*	82	34019.02	7374.65		12	42420.00	9242.62		29	52725.24	19465.28	28.696	0.001	b, c
Number recitation - Total time in direct order*	82	1829.60	605.14		12	2739.17	2020.92		29	2566.55	987.14	10.132	0.001	a, b
Number recitation - Total time in reverse order*	82	2993.05	1275.49		12	5245.00	4691.51		29	8898.62	8930.20	17.322	0.001	b
Picture search - Total time*	82	69939.16	32629.23		12	82961.67	55069.49		29	99100.38	54026.37	5.524	0.004	b
Boxes search - Total time*	82	53576.77	33330.10		12	91724.17	41731.51		29	106745.17	61017.45	18.820	0.001	a, b
Semantic word fluency - Total correct items	82	52.65	11.73		12	46.67	14.76		29	44.48	15.55	4.691	0.013	b
Semantic word fluency - perseverative errors	82	1.14	1.47		12	1.00	1.48		29	0.86	1.12	0.459	1.000	b
Word semantic fluency - Total efficiency	82	0.97	0.04		12	0.98	0.02		29	0.98	0.03	-	-	-
Day-night Stroop - Total time*	82	23732.93	9869.96		12	26155.83	6649.46		29	30733.45	9678.87	5.748	0.003	-
Abstract Stroop - Total time*	82	21498.05	9372.15		12	23426.67	4166.53		29	25129.66	4864.53	2.206	0.124	b
Digit span - Total score in direct order	82	12.40	4.09		12	9.75	3.57		29	8.03	2.37	15.870	0.001	b
Digit span - Total score in reverse order	82	9.11	2.99		12	7.75	3.19		29	5.86	2.31	13.898	0.001	b
List discrimination test - Total recognition score	82	19.12	1.97		12	19.33	0.78		29	18.14	2.63	2.705	0.089	-
List discrimination test - Total recency score	82	15.45	2.70		12	13.92	3.15		29	12.93	3.14	8.884	0.001	b
Tower of Hanoi - Total number of moves	82	10.35	5.37		12	8.92	3.20		29	7.97	2.57	2.930	0.061	-
Tower of Hanoi - Total number of rule violations	80	0.27	0.63		12	0.54	0.88		29	0.40	0.67	1.32	0.430	-
Bender Gestalt Test	82	31.29	5.22		12	30.42	4.81		29	26.17	9.04	7.121	0.001	b

9-HPT: Nine-hole Peg Test;

* The results are expressed in milliseconds; p: significance value associated with the main effect of group factor ANOVA analysis; LDT: List discrimination test. a: Differences post hoc between control group and HIV group; b: Differences post hoc between control group and AIDS group; c: Differences post hoc between HIV group and AIDS group.

Many of the differences were related to the control vs AIDS groups. This result is in agreement with findings in the literature that indicate more advanced stages of the disease are related to worse outcomes in neuropsychological evaluations. Regarding the differences between the control group and HIV group, the measures that differentiated the two groups were number recitation (Total time in direct order) and boxes search, both tasks related to processing speed. The only task that showed significant differences between the HIV vs AIDS groups was the task of picture naming (total time).

**Figure 1:**
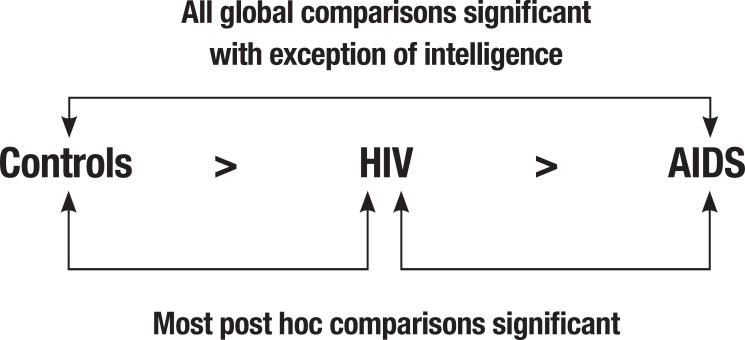
Summary interpretation of group comparisons. Global comparisons estimated with ANOVA and *post hoc* comparisons analyzed using Bonferroni's test.

In summary, HIV-infected children and adolescents exhibited lower performance on neuropsychological tasks than sociodemographically comparable typically developing controls while AIDS children presented lower performance than both these two groups (Figure 1). Motor and cognitive processing speed and executive function appeared to be the most discriminative domains, and HIV-infected individuals with more-advanced CDC disease stages (AIDS group) had lower performance levels and greater performance heterogeneity on the neuropsychological tasks.

**Effect sizes.** The accuracy of neuropsychological test scores in discriminating controls from clinical individuals (HIV + AIDS) was examined using Receiver Operating Characteristic (ROC) curve analysis. The results are shown in Table 3. Considering an area-under-the-curve of 0.70, seven measures of processing speed and executive function reliably discriminated between the control and clinical groups: number recitation in forward and reverse orders, color naming, visual search of boxes, picture naming, Tower of Hanoi (total number of moves), and 9-HPT (nondominant hand).

**Table 3:** ROC estimates for accuracy of neuropsychological tests in discriminating between controls and clinical group (HIV + AIDS).

Neuropsychological tests	AUC	Std. error	p	95% confidence interval	
				Lower	Upper
Number recitation - Total time in direct order	0.729	00.046	0.000	0.639	0.818
Number recitation - Total time in reverse order	0.838	00.038	0.000	0.764	0.913
Boxes visual search - Total time	0.835	0.036	0.000	0.764	0.906
Color naming - Total time	0.779	0.046	0.000	0.689	0.869
LDT - Recency score	0.303	0.052	0.000	0.202	0.404
Picture naming - Total time	0.842	0.037	0.000	0.768	0.915
Tower of Hanoi - Total number of moves	0.807	0.044	0.000	0.721	0.893
Digit Span - Total score in direct order	0.400	0.051	0.070	0.300	0.500
LDT - Recognition score	0.225	0.044	0.000	0.138	0.311
Santucci's copy drawing test - Total score	0.388	0.054	0.042	0.282	0.494
9-HPT -Total mean time of both hands	0.358	0.056	0.010	0.248	0.468
Abstract Stroop - Total time	0.675	0.053	0.002	0.572	0.778
9-HPT - Total mean time for dominant hand	0.727	0.046	0.000	0.636	0.818
Day-night Stroop - Total time	0.687	0.052	0.001	0.585	0.789
9-HPT -Total mean time for nondominant hand	0.714	0.048	0.000	0.620	0.809
Digit Span- Total score for reverse order	0.631	0.054	0.018	0.525	0.737
Tower of Hanoi - Total number of rule violations	0.233	0.047	0.000	0.140	0.325
Pictures search - Total time	0.577	0.055	0.161	0.469	0.686
Word semantic fluency - Total number of perseverative errors	0.628	0.055	0.021	0.519	0.737
Word semantic fluency - Total number of correct items	0.357	0.054	0.010	0.251	0.463
Raven - Percentile scores	0.320	0.049	0.001	0.225	0.415

AUC: Area under the ROC curve; LDT: List discrimination test; 9-HPT: Nine-hole peg test.

## DISCUSSION

The main goals of the present study were to investigate neuropsychological function in a cohort of Brazilian HIV/AIDS-infected individuals who were on HAART regimens. Compared with community-derived, sociodemographically pseudo-matched controls, AIDS patients exhibited lower performance on several cognitive measures. The differences were generally not greater than one standard deviation, showing a tendency of the clinical group to decline in several cognitive domains according to advanced stages of the disease. Psychomotor and cognitive speed and executive function tests revealed greater accuracy for discriminating the clinical sample from the control group, reflected by areas under the ROC curve that were greater than 0.70. A group of psychomotor/cognitive speed and executive function tests were also weakly correlated with the CDC stage of disease evolution. Thus, the observed neuropsychological impairments, although found to be more pronounced in participants with more advanced stages of the disease, did not correlate with the variable used (CDC stage). This may be because the current CDC neuropsychological indicator does not reflect actual losses incurred, since only clinical and immunological parameters for the definition of categories, without the presence of cognitive indicators for the implementation, are used.

Considering the dispersion of both ages and neuropsychological scores in the clinical group, the consistency of the results is noteworthy (Table 2). Remarkably, although they were relatively lower, Raven z-scores in the HIV/AIDS group did not surpass one standard deviation below the controls' means, and the differences were not statistically significant. The relatively mild nature of neuropsychological impairment and preservation of intelligence is consistent with data reported from developed countries^13-15,17^ but diverge from common observations of more severe neuropsychological impairment in children and adolescents from developing countries.^42,43^ This discrepancy can most likely be attributed to successful AIDS treatment program implementation in Brazil since 1996.44 Moreover, the disparities may be due to the different measures of intelligence used in the studies.^42^ The measure of intelligence used was the Kaufman assessment battery for children (K-ABC) and the differences found were related to the areas of sequential processing, simultaneous processing, mental processing and nonverbal skills. Thus, regarding the other cognitive measures employed, no differences between groups were found, including for intelligence. In the study,^43^ the observed changes occurred mainly in verbal skills measured, without changing the performance of execution tasks. This raises questions over the ability of HAART to protect/improve the cognition of the HIV-infected children. Data on the protective effects of HAART on cognition in HIV-infected persons are not a consensus in the scientific literature. A recent meta-analysis^49^ showed only moderate effects on the conservation and improvement of cognitive performance, whereas some other studies have shown no significant effects.^50^

The lack of specificity of the results also warrants consideration. Although accuracy estimates were higher for psychomotor and cognitive processing speed and executive function tasks, practically all of the group comparisons reached statistical significance, with the exception of the Tower of Hanoi and semantic word fluency task. These results suggest that although psychomotor, cognitive processing speed and executive function represent important domains of impairment in pediatric HIV/AIDS, other areas, such as visuospatial memory and language function, are also frequently impaired. Consistent with our findings,^45^ results indicate that although relatively subtle, neuropsychological impairment in adequately treated HIV/AIDS-infected children and adolescents may be broader and not restricted to functions subserved by fronto-striatal reentrant connections.^22^

In addition to the impact of neuropsychological impairment on school performance, other issues that merit clarification in future research are related to the neural basis and specificity of such impairments. The data are still scarce but suggest that neuropsychological deficits in adequately treated HIV/AIDS-infected children and adolescents extend beyond the subcortical white matter profile. This issue can be addressed only in the context of neuroimaging studies and larger samples that allow factor and cluster analyses.^22^

Some of the main strengths of the present study deserve qualification. The study design was neuropsychologically conceived, generating data that may contribute to the elucidation of the neural basis of subtle cognitive impairments observed in adequately treated HIV/AIDS-infected children and adolescents. Important in the present context is the sociodemographically comparable control group of normally developing individuals that was derived from a larger community-derived pool of participants. The sample size was also considerably larger than, or at least equivalent to, the sample sizes presented in other similar reports.^13-17^

The limitations of the present study should also be mentioned. The number of HIV-infected individuals who did not develop AIDS was relatively small, restricting comparisons between this group and AIDS patients or typical controls. Therefore, we were unable to evaluate the impact of subtle disclosed neuropsychological impairment on this important outcome measure. This study corroborates recent findings on cognitive related losses, especially in the fields of executive functions and processing speed.

The choice of executive function tests should also be optimized by incorporating more pure and theoretically-oriented measures according to the trifactorial model^25,26^ Larger samples should allow analyses of the factor structure of executive function to identify domains of any selective impairment. Ideally, dependence analysis should also be performed to elucidate the role of processing speed in cognitive development,^46^ intelligence,^47^ and working memory.^48^

This study is expected to foster discussion about the cognitive losses of HIV infection in the Brazilian context and points to the need to invest in assessment protocols implemented within treatment units. Given the nature and severity of losses, variables can be of interest in future outcomes, particularly as regards the academic skills and joining the labor market. The differences cited here reveal preliminary evidence that the cognitive losses occur as the disease progresses and underscore the need to plan policies for specific interventions for reducing their affect and to develop compensatory strategies that can minimize their impact on daily life.
